# Quantification of Polyfunctional Thiols in Wine by HS-SPME-GC-MS Following Extractive Alkylation

**DOI:** 10.3390/molecules200712280

**Published:** 2015-07-06

**Authors:** Lauren E. Musumeci, Imelda Ryona, Bruce S. Pan, Natalia Loscos, Hui Feng, Michael T. Cleary, Gavin L. Sacks

**Affiliations:** 1 Department of Food Science, Cornell University, Stocking Hall, 411 Tower Road, Ithaca, NY 14853, USA; E-Mails: lem248@cornell.edu (L.E.M.); ir45@cornell.edu (I.R.); 2 E. & J. Gallo Winery, 600 Yosemite Boulevard, Modesto, CA 95354, USA; E-Mails: Bruce.Pan@ejgallo.com (B.S.P.); Natalia.Loscos@ejgallo.com (N.L.); Hui.Feng@ejgallo.com (H.F.); Mike.Cleary@ejgallo.com (M.T.C.)

**Keywords:** wine, polyfunctional thiols, extractive alkylation, *Vitis labruscana*

## Abstract

Analyses of key odorous polyfunctional volatile thiols in wines (3-mercaptohexanol (3-MH), 3-mercaptohexylacetate (3-MHA), and 4-mercapto-4-methyl-2-pentanone (4-MMP)) are challenging due to their high reactivity and ultra-trace concentrations, especially when using conventional gas-chromatography electron impact mass spectrometry (GC-EI-MS). We describe a method in which thiols are converted to pentafluorobenzyl (PFB) derivatives by extractive alkylation and the organic layer is evaporated prior to headspace solid phase microextraction (HS-SPME) and GC-EI-MS analysis. Optimal parameters were determined by response surface area modeling. The addition of NaCl solution to the dried SPME vials prior to extraction resulted in up to less than fivefold improvement in detection limits. Using 40 mL wine samples, limits of detection for 4-MMP, 3-MH, and 3-MHA were 0.9 ng/L, 1 ng/L, and 17 ng/L, respectively. Good recovery (90%–109%) and precision (5%–11% RSD) were achieved in wine matrices. The new method was used to survey polyfunctional thiol concentrations in 61 commercial California and New York State wines produced from *V. vinifera* (Riesling, Gewürztraminer, Cabernet Sauvignon, Sauvignon blanc and non-varietal rosé wines), *V. labruscana* (Niagara), and *Vitis* spp. (Cayuga White). Mean 4-MMP concentrations in New York Niagara (17 ng/L) were not significantly different from concentrations in Sauvignon blanc, but were significantly higher than 4-MMP in other varietal wines.

## 1. Introduction

Volatile thiols are well known for possessing low sensory detection thresholds and contributing to the odor of a range of foodstuffs, including wine [1]. Although low-molecular weight thiols like methyl mercaptan are often associated with wine off-aromas [2], higher molecular weight polyfunctional thiols can contribute to the desirable varietal character of many wines, most notably Sauvignon blanc [3]. These polyfunctional thiols are largely lacking from winegrapes (*Vitis vinifera*) and are primarily formed during fermentation by microbial metabolism of non-volatile precursors, e.g., S-glutathione and S-cysteine conjugates [1]. While several supra-threshold polyfunctional thiols have been identified in wine, three are reported to be of particular relevance: 4-mercapto-4-methyl-2-pentanone (4-MMP, detection threshold = 0.8 ng/L), 3-mercaptohexanol (3-MH, 60 ng/L), and 3-mercaptohexyl acetate (3-MHA, 4.2 ng/L), described as having box tree, grapefruit, and passion fruit aromas, respectively [3,4].

Analyses of polyfunctional thiols in wine is challenging due to their low concentrations, susceptibility to oxidation, and poor chromatographic behavior [1,5]. Direct analysis of free thiols following a pre-concentration step such as solid phase extraction (SPE), solid-phase microextraction (SPME), stir-bar sorptive extraction (SBSE), or related techniques prior to gas-chromatography electron impact mass spectrometry (GC-MS) have been reported [6,7], but analytical detection limits are well above sensory thresholds. As a result, the majority of studies on polyfunctional thiols in wines have utilized more specialized methods to remove interferences and/or improve GC-MS response.

One general approach for pre-concentration and clean-up of polyfunctional thiols in wine involves performing mercurobenzoate derivatization followed by anion exchange chromatography [4]. Analytes are then released by addition of a competing thiol, and quantified by gas chromatography mass spectrometry (GC-MS). The drawbacks of this approach are that it utilizes toxic Hg salts, risks sample oxidation, and requires large sample sizes (1–2 L wine) to achieve acceptable limits of detection [8]. 

A second approach is to derivatize thiols prior to GC-MS analysis to improve their stability, chromatographic behavior, and mass spectrometric response. Several reports have reported that pentafluorylbenzyl (PFB) derivatives of wine thiols yields good linearity and sub-sensory threshold detection limits when GC-negative ion chemical ionization-MS (GC-NICI-MS) is used as a detector [9,10,11,12]. Several sample preparation variants have been employed prior to GC-NICI-MS, including liquid-liquid extraction (LLE) followed by derivatization and liquid injection [9]; on-cartridge SPE derivatization followed by wash steps, elution with organic solvent and liquid injection [10]; and on-cartridge SPE derivatization followed by wash steps, elution, evaporation of the organic solvent, and SPME of the dried down residue [12]. For these methods, methoximation of the ketone group of 4-MMP prior to derivatization has been suggested to increase the PFB derivatization yield [10].

However, NICI-MS is not routinely available to most labs, and analysis of PFB-thiol derivatives by the more commonly available electron impact ionization MS (EI-MS) results in severe losses of sensitivity. In one report, a detection limit of 30 ng/L for 3-MH was achievable using GC-EI-MS detection after an initial LLE of a 200 mL wine sample into pentane followed by back-extraction into alkaline aqueous solution, PFB derivatization, and headspace solid phase microextraction (HS-SPME), [13]. Quantification of 4-MMP and 3-MHA was not reported. By comparison, a 2 ng/L detection limit was achieved for a 10 mL wine sample using on-cartridge SPE derivatization followed by GC-NICI-MS [10]. Ethyl propiolate derivatization followed by GC-EI-MS detection has also been described but detection limits were 10-fold above sensory threshold for 4-MMP and 3-MHA [14]. A SPME-GC-MS/MS method for 4-MMP following methoxime derivatization is reported to have <1 ng/L detection limits [15], but requires the availability of tandem MS, and the method’s suitability for other thiols is unclear. Promising methods based on derivatization of thiols with 4,4ʹ-dithiodipyridine [16] or *o*-phthaldialdehyde [17] were recently described, but requires the availability of an LC-MS/MS for quantification. In summary, current methods for polyfunctional thiol analyses in wine suffer from poor sensitivity or else require intensive sample preparation, large sample sizes, toxic reagents, and/or instrumentation that may not be routinely available. 

This paper describes the optimization and validation of a new method utilizing SPME-GC-EI-MS for quantification of PFB derivatives of three polyfunctional thiols, 4-MMP, 3-MH, and 3-MHA, with detection limits at or near their respective sensory thresholds. Beyond the advantages inherent to using the more common GC-EI-MS, the method also simplifies extraction, derivatization, and clean-up steps based on extractive alkylation prior to evaporation of the organic layer and HS-SPME. We also demonstrate that improvements in HS-SPME recovery can be realized by addition of –NaCl solution to the dried organic residue. The new method was then used for measurement of the three thiols in California and New York State wines in *V. vinifera* wines as well as wines from interspecific hybrid grapes (*Vitis* spp. and *V. labruscana*).

## 2. Results and Discussion

**Figure 1:**
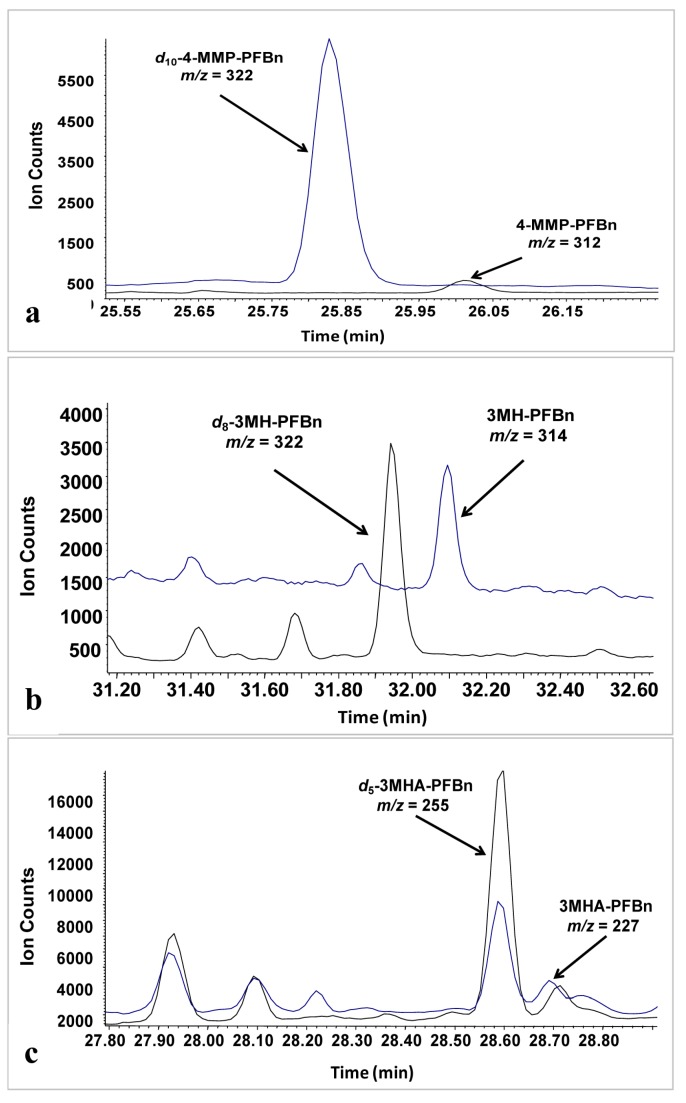
Typical chromatogram obtained in selective ion monitoring (SIM) mode: (**a**) White wine spiked with 10 ng/L 4-MMP and 540 ng/L *d*_10_-4-MMP; (**b**) White wine spiked with 25 ng/L 3-MH and 440 ng/L *d*_8_-3-MH; (**c**) White wine spiked with 60 ng/L 3-MHA and 400 ng/L *d*_5_-3-MHA.

Analysis of key wine polyfunctional thiols (3-MHA, 3-MH, 4-MMP) in underivatized form is challenging, and reports that have achieved detection limits below sensory threshold have required the use of organomercuric reagents to achieve a high degree of selective pre-concentration. Alternate quantification schemes for thiols involving derivatization prior to analysis exist [18]. We chose to develop an optimized method based on pentafluorobenzylbromide (PFBBr) derivatization, as this approach has previously been demonstrated to be effective for the key wine polyfunctional thiols [9,10,11,19]. Representative chromatograms for wine samples analyzed by the optimized method are shown in Figure 1.

### 2.1. Optimization of Extractive Alkylation Conditions

**Figure A1:**
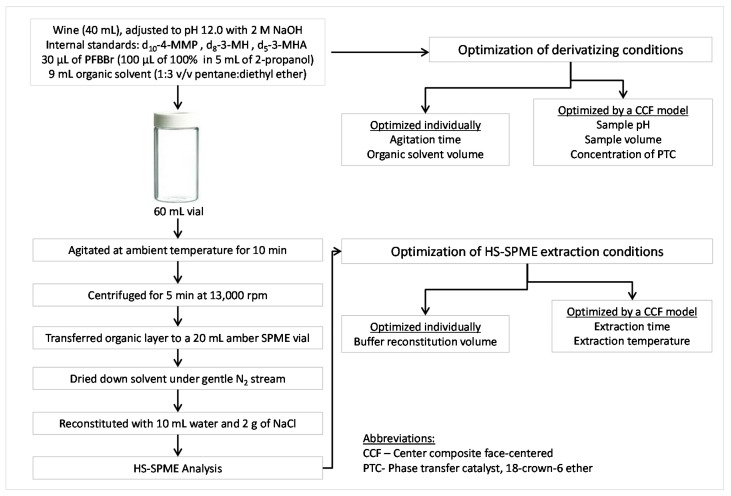
Schematic overview of thiol extraction and quantification, including optimization steps.

A schematic overview of the thiol analysis method is shown in Figure A1, and steps subjected to optimization are underlined. To simplify sample preparation and reduce the risk of thiol oxidation before derivatization, we investigated the use of extractive alkylation, in which derivatization and extraction occur in a single step. Under these conditions, PFBBr derivatization of the thiol involves nucleophilic substitution by the thiolate in a heterogenous system, and the reaction typically proceeds more rapidly in the presence of phase-transfer catalysts like 18-crown-6-ether [20]. Although extractive alkylation with PFBBr is widely used [20], only one group has reported an (unsuccessful) attempt to perform extractive alkylation of thiols in wine but no details were presented as to why the procedure did not work [9].

As a first step, we optimized conditions for extractive alkylation. Optimization experiments are typically performed with the goal of maximizing analyte signals, for example, in determining optimal SPME extraction temperature and extraction time for analysis of 3-alkyl-2-methoxypyrazines in grape musts [21]. In our method, we observed up to tenfold variation in both analyte signal and background noise across runs for replicate analyses during optimization experiments. The reason for this variation was unclear, but because the signal intensity and noise intensity were well correlated, it may be due to variation in the drying step. To overcome this problem, we attempted to minimize the detection limit (LOD) in our optimization experiments, where LOD was defined as the minimum concentration necessary to achieve 3 times the noise limit.

#### 2.1.1. Reaction Time and Organic Solvent Volume

**Figure A2:**
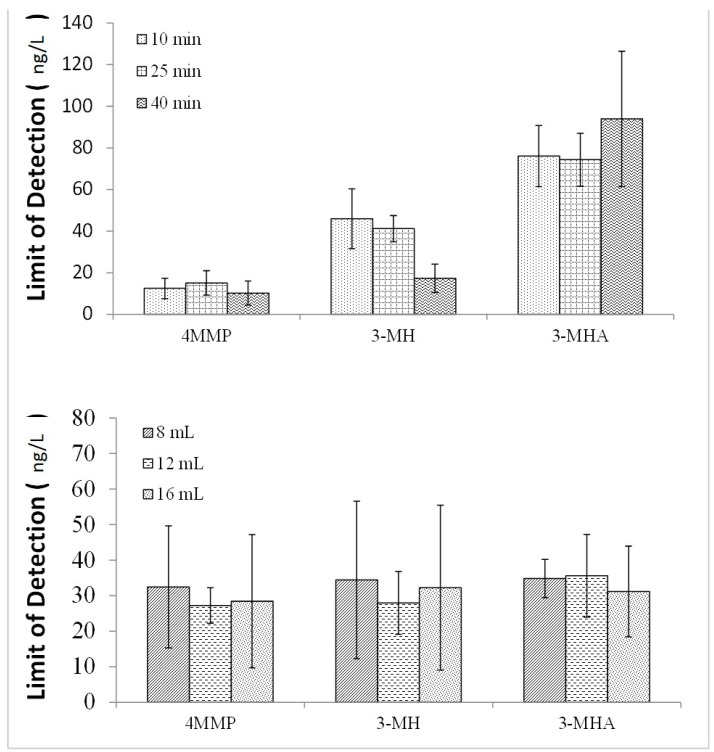
Effects of agitation time (**top**) and volume of organic solvent (**bottom**) on detection limits obtained for each thiol. Error bars represent ± 1 standard deviation for 3 replicates.

The effects of reaction time and organic solvent volume on extractive alkylation were investigated individually. No significant differences in LODs for 4-MMP and 3-MHA were obtained for reaction times of 10, 25, and 40 min at room temperature (Figure A2). For 3-MH, the LOD decreased significantly by about a factor of two when reaction time was increased from 10 min to 40 min (*p* < 0.05). Because the detection limit for 3-MH was below sensory threshold even without further optimization, we chose to use a 10 min reaction time for expedience.

The volume of the pentane:diethyl ether organic solvent was found to have no significant impact on LOD when varied over 8 to 16 mL (Figure A2). This likely indicates that the PFB derivatives are sufficiently non-polar to partition nearly completely into the organic phase for the range of organic volumes used. A volume of 9 mL was selected for the optimized protocol for convenience, although it is possible that even less solvent could have been used.

#### 2.1.2. Sample pH, Sample Volume, PTC Concentration

**Figure 2:**
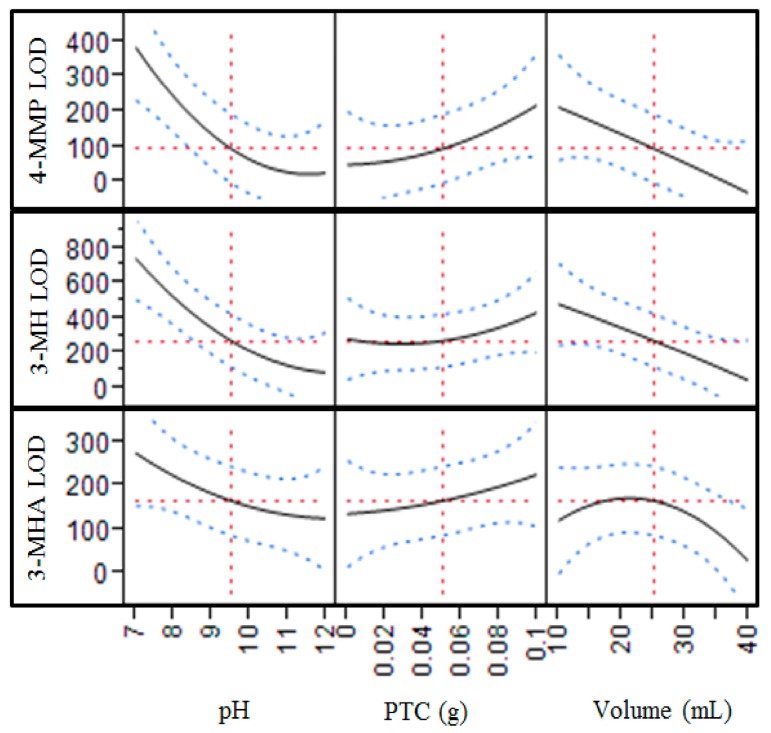
Effects of pH, PTC concentration, and sample volume on limits of detection for each thiol. The optimization was performed using a multifactorial CCF model. The red dotted lines show the mean values within a factor (**vertical lines**) or for a thiol detection limit (**horizontal lines**). The blue dotted lines represent the error bars.

Optimal values for pH, sample volume, and PTC concentration were evaluated concurrently in a multifactor fractional factor model (center composite face, CCF), and results are shown for each parameter in Figure 2. LOD was significantly affected by pH (*p* < 0.01 for all thiols), with the lowest LOD values achieved under the most alkaline conditions (pH = 12). As expected, LODs also decreased with increasing wine volume from 10 to 40 mL (*p* < 0.01 for 4-MMP and 3-MH; *p* < 0.1 for 3-MHA). We also determined that signal would continue to scale up proportionally when 160 mL of wine was used instead of 40 mL (data not shown). For convenience, the smaller volume of 40 mL was chosen for the optimized protocol, but improved LOD are attainable by using greater sample volumes. Significant interaction terms (*p* < 0.05) were observed for pH × volume, pH × catalyst and catalyst × volume, although this information was not further used for method optimization. Surprisingly, the presence of the PTC (18-crown-6 ether) did not improve LODs for any of the thiols (Figure 2). It is not clear why the reaction proceeded efficiently even without a PTC, but as a result no PTC was used in the optimized protocol. Mateo-Vivracho *et al*. have previously reported an improvement in PFBBr derivatization following methoximation of 4-MMP [10]; however, because we observed acceptable sensitivity for 4-MMP without this step, we did not explore methoximation.

### 2.2. Optimization of HS-SPME Conditions

A previous report using GC-NICI-MS reported detection limits <10 ng/L for all three thiols following a 4-fold concentration by solvent extraction, PFBBr derivatization, and large volume (20 μL) injections [9]. Preliminary investigations of liquid injection of the organic phase by our group with roughly the same mass injected (25-fold concentration factor by evaporation of organic phase, 3 μL injection) and GC-EI-MS detection resulted in unacceptable detection limits, approximately 1 μg/L for each thiol (data not shown).

As an alternative pre-concentration approach to partial solvent evaporation, we next explored completely removing the organic solvent prior to SPME extraction. This approach had been successfully applied by another group in analysis of thiol-PFB derivatives using GC-NICI-MS [12]. A two-phase PDMS/DVB fiber was selected, as this was reported to give better peak shape characteristics than a three-phase polydimethylsiloxane/divinylbenzene/Carboxen (PDMS/DVB/CARB) fiber [13]. As a two centimeter PDMS/DVB fiber is not commercially available, a one centimeter PDMS/DVB fiber was chosen for the optimization study.

#### 2.2.1. Reconstitution Volume

**Figure 3:**
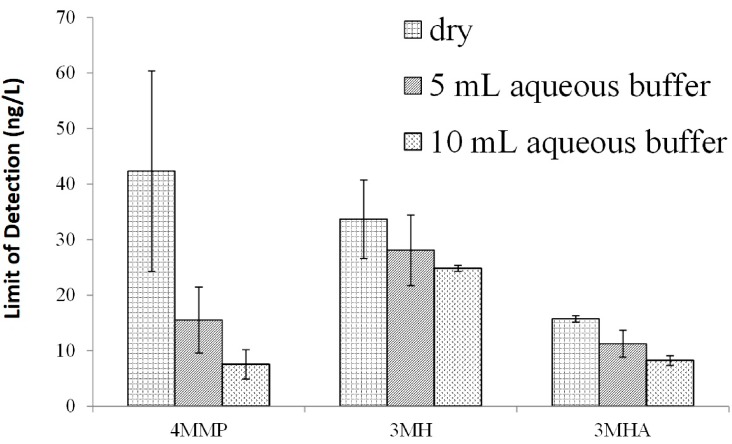
Detection limits for thiols obtained for dry vials or for vials reconstituted with 5 or 10 mL of aqueous buffer. Error bars represent ± 1 standard deviation for two replicates.

We investigated if the addition of aqueous NaCl to the dried-down SPME vials could increase signal and decrease detection limits, which has been previously reported for quantification of pesticide residues in soil [22]. We observed a significant decrease in LOD for all three thiols upon addition of aqueous NaCl (*p* < 0.1 for dry vial *vs.* 10 mL buffer), as shown in Figure 3. The largest effect was observed for 4-MMP, where the LOD decreased by five-fold when 10 mL of NaCl solution were added, as compared to the control with no added solution. A potential explanation for this effect is that the aqueous solution used for reconstitution can compete for active sites on the glass SPME vial, decreasing adsorptive losses of the thiol-PFB analytes. Alternatively, the presence of water could alleviate hot spots of the SPME vial during extraction, preventing thermal degradation of the analytes.

#### 2.2.2. HS-SPME Extraction Time and Temperature

**Figure 4:**
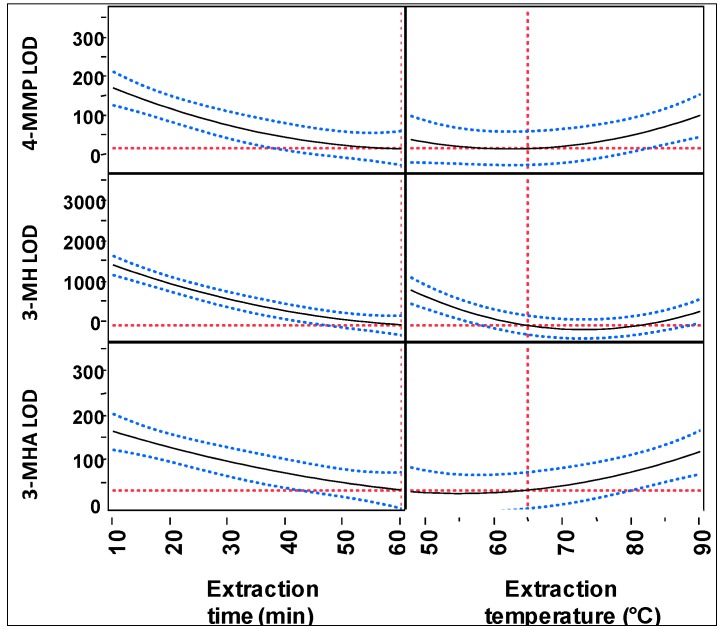
Effect of extraction temperature and extraction time on detection limits for thiols. The optimization was performed using a multifactorial CCF model. The red dotted lines show the mean values within a factor (**vertical lines**) or for a thiol detection limit (**horizontal lines**). The blue dotted lines represent the error bars.

Optimal values for SPME extraction temperature and time were evaluated concurrently in a multifactor CCF model, and results are shown for both parameters in Figure 4. SPME extraction time had a significant effect for all three thiols (*p* < 0.001), with the longest extraction time of 60 min decreasing detection limits by two to three orders of magnitude. Extraction temperature also had a significant effect on detection limits for the three thiols (*p* ≤ 0.1 for 4-MMP and 3-MHA; *p* < 0.0001 for 3-MH). The extraction temperature chosen for the optimized method was 70 °C.

### 2.3. Figures of Merit

#### 2.3.1. Linearity and Limits of Detection

Linearity was evaluated by addition of standards to a model white wine at eight levels. The observed linearity was excellent for the three thiols (*r*^2^ > 0.99, Table 1). The linear range for 3-MH and 4-MMP covered the full range of standards (6–20,000 ng/L). The linear range for 3-MHA (63–6325 ng/L) was more limited, as the two lowest concentrations were at or below the detection limit, and the highest concentration interfered with the deuterated standard trace.

**Table 1:** The observed concentrations at varying levels of 4-mercapto-4-methyl-2-pentanone (4-MMP), 3-mercaptohexanol (3-MH), and 3-mercaptohexylacetate (3-MHA) resulting from standard addition.

Spike Level (ng/L)	ng/L of 4-MMP	ng/L of 3-MH	ng/L of 3-MHA
6	8.0 (±0.3)	4.1 (±0.1)	n.d.
20	19 (±0.7)	19 (±2.1)	n.d.
63	50 (±3.2)	66 (±3.3)	72 ( ± 4.9)
200	179 (±12.2)	202 (±11.3)	178 (±10.0)
632	525 (±50.1)	722 (±5.7)	589 (±26.7)
2000	1792 (±133.2)	2245 (±16.4)	2080 (±42.5)
6325	5848 (±219.4)	6335 (±88.8)	6301 (±218.5)
20,000	20826 (±2599.2)	19652 (±191.0)	n.d.
σ_i_, ng/L	0.31	0.33	5.8
LOD, ng/L	0.9	1.0	17.3
*r*^2^	0.996	0.998	0.999
Lack of Fit Test (*p* value)	0.9946	0.0741	0.8969

Standard deviations (*n* = 2) were stated in parenthesis. Abbreviations: σ_i_ = signal independent background noise, LOD = Limit of detection, n.d. = not detected.

Method detection limits were determined by standard addition of thiols to a model wine and are reported in Table 1. Signal-independent noise and limits of detection were calculated based on all analytical replicates using Pallesen’s method [23]. The limits of detection for 4-MMP (0.9 ng/L) and 3-MH (1 ng/L) compared well to their respective sensory thresholds, 0.8 ng/L and 60 ng/L. The LOD for 3-MHA was somewhat higher than its sensory threshold (17 *vs.* 4.2 ng/L) although still below concentrations reported in many Sauvignon blanc wines (mean = 29–516 ng/L, depending on region [24]) as well as other white wines [3]. The high LOD of 3-MHA in comparison to those achieved for 4-MMP and 3-MH was due largely to the lack of characteristic, interference-free ions for 3-MHA-PFB.

#### 2.3.2. Accuracy and Precision

Accuracy was evaluated by recovery spikes of the three thiols into a commercial Pinot grigio wine at high (500 ng/L) and low (60 ng/L) concentrations of each thiol. The native concentrations of thiols in the wine were: 3-MH (228 ng/L), 3-MHA (<LOD), and 4-MMP (2.1 ng/L). Recovery was acceptable, and ranged from 90%–109% of the expected values (Table 2). The calibration curves were generated in model wine, indicating that the isotopically labeled standards effectively compensated for any matrix differences between the real and model wine systems. Precision was evaluated by spikes at low (10 ng/L 4-MMP; 25 ng/L 3-MH; 25 ng/L 3-MHA) and high concentrations (100 ng/L 4-MMP; 200 ng/L 3-MH; 100 ng/L 3-MHA) of each thiol into the same Pinot grigio. Relative standard deviations were 5%–7% for the high concentration spikes and 7%–11% for the low concentration spikes, with the worst performance for the low concentration 3-MHA spike.

**Table 2:** Recovery and precision experiments.

Analyte	Recovery (%)		RSD (%)	
	Low Level	High Level	Low Level	High Level
4-MMP	104.9	108.7	9.8	6.6
3-MH	102.6	90.5	6.9	5.4
3-MHA	90.2	100.5	11.1	5.6

Recovery: samples spiked at low level (60 ng/L 4-MMP, 3-MH, 3-MHA); high level (500 ng/L 4-MMP, 3-MH, 3-MHA). RSD: relative standard deviation of 5 samples spiked at low level (10 ng/L 4-MMP; 25 ng/L 3-MH; 25 ng/L 3-MHA); high level (100 ng/L 4-MMP; 200 ng/L 3-MH; 100 ng/L 3-MHA).

### 2.4. Comparison of Current Method to Existing GC-MS Methods for Wine Polyfunctional Thiol Analysis

Literature methods that rely on a low selectivity extraction step (e.g., SPE, SPME) to pre-concentrate underivatized wine polyfunctional thiols (3-MH, 3-MHA, 4-MMP) prior to GC-EI-MS have not achieved detection limits below sensory thresholds [6,7]. Quantification of these thiols below sensory threshold can be achieved using organomercuric reagents and anion exchanges for selective pre-concentration [4], but the approach has been criticized for needing large sample volumes and hazardous reagents [10,11].

More recent reports have achieved detection limits below sensory thresholds by preparing PFBBr derivatives of the thiols prior to GC-NICI-MS detection. The detection limits of these methods are generally better than what we have achieved with our current report: for example Mateo-Vivaracho, *et al.* reported LODs of 0.1 ng/L, 2 ng/L, and 0.3 ng/L, for 4-MMP, 3-MH, and 3-MHA, respectively, as compared to 0.9 ng/L, 1 ng/L, and 17 ng/L in our work. Correcting for the fact that this earlier report used a 6 mL wine sample compared to 40 mL in our work, NICI-MS provides about a 1–3 order of magnitude improvement in detection limits compared to EI-MS for PFB derivatives, comparable to values reported for other compound classes [25]. 

However, an advantage of our current method is that EI-MS is more widespread than NICI-MS in laboratories. One other group has utilized GC-EI-MS for quantification of the 3-MH-PFB derivative, although they did not report characterization of 3-MHA and 4-MMP [13]. In that report, 3-MH was initially extracted into pentane and back extracted into aqueous alkaline buffer prior to derivatization. Similar to our approach, the 3-MH-PFB derivative was extracted by SPME prior to GC-EI-MS. We achieved a detection limit of 1 ng/L for a 40 mL wine sample, as compared to 30 ng/L for a 200 mL wine sample in the previous report. The most notable difference between the two reports is that our current work utilized a dry-down of the organic layer step prior to reconstitution and HS-SPME, as opposed to the back-extraction step in the other report. Potentially, this dry-down step removed lower boiling volatiles that could compete with the thiol-PFB derivatives for absorptive sites on the SPME fiber. While we observed a considerable enhancement in S/N when aqueous NaCl solution was added to our dried-down SPME vials, this should have been comparable to the conditions in the other report for alkaline back-extraction, and is thus unlikely to account for the differences. Other potential explanations for the enhanced sensitivity of our current report are more optimal derivatization or HS-SPME conditions, or decreased losses during sample preparation due to fewer work-up steps. GC-EI-MS has also been reported for quantification of ethyl propiolate derivatives of thiols, but detection limits were well above sensory thresholds for 4-MMP and 3-MH [14].

Beyond using EI-MS, another advantage of our extractive alkylation method is that it requires fewer clean-up steps than many other methods that have analyzed PFB derivatives. For example, one report describes an initial loading of the sample onto an SPE cartridge followed by multiple wash steps, introduction of a catalyst and derivatizing reagents, addition of mercaptoglycerol to remove excess PFBBr, and, finally, elution of the derivatized thiol with organic solvent [12]. The organic phase is then evaporated in a SPME vial prior to HS-SPME analysis, similar to our current work. Other reports also use multiple LLE or SPE clean-up steps, often prior to derivatization [9,10,13]. By comparison, the extractive alkylation described here can be performed in a single step.

### 2.5. Quantification of Polyfunctional Thiols in Commercial Wines

Using the optimized method, the three polyfunctional thiols were measured in 31 California wines and 30 commercial New York State wines representing seven wine types: four *V. vinifera* (Gewurztraminer, Riesling, Sauvignon blanc, Cabernet Sauvignon), one non-varietal *V. vinifera* (rosé blends), one *V. labruscana* (Niagara), and one *Vitis* spp. (Cayuga White). Results are summarized in Table 3. The only thiol detected in all wine samples was 3-MH, and concentrations were significantly higher in the *vinifera*-based wines than the interspecific hybrid-based wines (Cayuga White and Niagara). However, 3-MH concentrations were not significantly higher in Sauvignon blanc wines than the other white varietal wines, unlike results reported elsewhere [3].

**Table 3:** Concentrations of volatile polyfunctional thiols in California and NY wines.

Variety	Region	Concentration (ng/L)		
		4-MMP	3-MH	3-MHA
*Cayuga White*	Finger Lakes, NY (*n* = 5)	<LOD	195 ± 38	<LOD
*Niagara*	Finger Lakes, NY (*n* = 5)	18 ± 13	230 ± 159	<LOD
*Riesling*	Finger Lakes, NY (*n* = 5)	2.3 ± 2.5	569 ± 334	<LOD
*Gewürztraminer*	Finger Lakes, NY (*n* = 5)	<LOD	373 ± 134	<LOD
*Rosé*	Finger Lakes, NY (*n* = 5)	<LOD	296 ± 116	^a^
*Sauvignon blanc*	Finger Lakes, NY (*n* = 5)	27 ± 8	446 ± 154	^a^
	Napa Valley, CA (*n* = 4)	44 ± 22	438 ± 87	^b^
	Sonoma County, CA (*n* = 4)	43 ± 18	712 ± 342	^b^
	Central Coast, CA (*n* = 3)	50 ± 25	835 ± 137	^b^
*Cabernet Sauvignon*	Napa Valley, CA (*n* = 8)	<LOD	765 ± 396	57 ± 16
	Sonoma County, CA (*n* = 5)	<LOD.	405 ± 106	60 ± 21
	Central coast, CA (*n* = 6)	<LOD	498 ± 113	67 ± 24
	Lodi (*n* = 1)	<LOD	396	46

^a^ Only one sample had detectable values: Rose = 52.3 ng/L and Sauvignon blanc = 33.8 ng/L. ^b^ Interference on quantifier ion prevented accurate quantification.

Mean 3-MH concentrations were lower in New York and California Sauvignon blanc (less than 1000 ng/L) than reports from other regions. For example, a survey of commercial Sauvignon blanc wines from Australia, New Zealand, France, and South Africa reported mean concentrations ranging from 1700 to 7100 ng/L [24]. Mean concentrations of 3-MH in Sauvignon blanc in our survey were also lower than what was reported in an earlier survey of US Sauvignon blanc, ~2000 ng/L for eight wines over 3 vintages [24], although the lack of specific information regarding wine region and large standard deviations (~100%) in the previous report makes direct comparison challenging. In contrast, mean 3-MH in New York Gewürztraminer and Riesling (373 and 579 ng/L, respectively) were in the middle of ranges reported for Australian sources of these varietal wines [26]. 3-MH in the California Cabernet Sauvignon was within the broad range reported for Bordeaux red wines previously (10–5000 ng/L) [27]. The contribution of volatile thiols like 3-MH to red wines has not been well studied, but the mean values reported in this study (396–765 ng/L) are comparable to other non-Sauvignon blanc white wines, both in the current study and elsewhere [26].

The reasons for lower concentrations of 3-MH in the California and New York State Sauvignon blanc in this study as compared to other reports is unclear, as several factors are known to affect final 3-MH concentrations. For example, wine grapes are typically hand-harvested in the Finger Lakes and Napa, and it is reported that hand harvesting grapes results in lower 3-MH precursor concentrations and eventually lower 3-MH in wines as compared to machine harvesting [28]. However, this argument would not be appropriate for California Central Coast where machine harvesting is common. Other factors reported to affect final 3-MH concentrations include vineyard nitrogen availability, yeast assimilable nitrogen, abiotic stresses, disease pressure, oxygen status during maceration, and post-fermentation oxidation [1], and a recent study has reported modest correlations of over 20 grape metabolites (amino acids, fatty acids, *etc.*) with wine 3-MH concentrations [29]. Discerning which of these factors would lead to low 3-MH concentrations in Sauvignon blanc and Gewürztraminer samples in this study is not possible. 

It was found that 3-MHA was only detectable in two out of the 30 New York wines evaluated; one rosé wine, and one Sauvignon Blanc wine. Formed by partial enzymatic acetylation of 3-MH during fermentation [30], 3-MHA will decrease during storage due to acid-catalyzed hydrolysis [31]. As a result, average 3-MHA values are reported to be 10%–20% the concentration of average 3-MH values in Sauvignon blanc [29], and can be much lower [24]. Since the average 3-MH concentration across New York State wines in this study was 195 to 569 ng/L, and many of the wines were 2–3 years old at the time of analysis, it is unsurprising that 3-MHA was generally below the 17 ng/L detection limit of the method. Additionally, New York State grapes tend to be low in α-amino acids as compared to other regions [32], and several of these amino acids have been correlated with lower 3-MHA/3-MH ratios [29]. Furthermore, 3-MHA could not be quantified in the California Sauvignon blanc samples due to an unknown interference on the quantifier ion, which could not be identified by library search. However, 3-MHA could be detected in California Cabernet Sauvignon, and mean values across sub-regions (46–67 ng/L) were approximately 10% of 3-MH concentrations. Similar to 3-MH, there is a lack of analytical data regarding 3-MHA in red wines, but values are within the range estimated for Bordeaux red wines, 1–200 ng/L [27]. 

In contrast to 3-MH and 3-MHA, mean 4-MMP concentrations in both New York (27 ng/L) and California (44–50 ng/L) Sauvignon blanc wines were greater than the mean values (5–10 ng/L) reported for Sauvignon blanc from most other wine regions [24]. The highest reported mean value for 4-MMP in this previous survey was for the Wairarapa region of New Zealand (19 ng/L), but this region also had order of magnitude higher concentrations of 3-MH. In addition, 4-MMP was not detectable in other *V. vinifera* wines, except for trace concentrations (mean = 2.5 ng/L) in NY State Riesling wines.

To our knowledge, this is the first report of thiol concentrations in any American wines from specific wine regions, rather than the general category of US wine [24]. The study also included the first analysis of thiols in wines produced from interspecific hybrids-Niagara and Cayuga White. The former contains *V. labrusca* parentage, and the latter is a complex interspecific hybrid with mixed parentage. The 3-MH concentrations in Cayuga White were significantly lower than in *vinifera* wines, and the other two thiols were not detectable. However, concentrations of 4-MMP in New York Niagara (mean = 18 ng/L) did not differ significantly from New York Sauvignon blanc (*p* > 0.05), and were significantly higher than all other varietal wines (*p* < 0.05). The “foxy” smelling *o*-aminoacetophenone and methyl anthranilate have previously been implicated in contributing to Niagara juice and wines [33,34]. Because 4-MMP exceeded sensory threshold by an order of magnitude in the majority of Niagara wines, 4-MMP may also be important to the varietal character of Niagara wines, but confirmation by sensory studies was not part of this study.

## 3. Experimental Section

### 3.1. Chemical Reagents and Standards

Sodium chloride (NaCl), sodium hydroxide (NaOH), 18-crown-6-ether, pentane, 2-propanol, diethyl ether, ethanol, and the unlabeled standards, 3-MH, 4-MMP, and 3-MHA, were purchased from Sigma Aldrich (Allentown, PA, USA) at the highest available purity. The deuterated internal standards, ^8^[H_2_]-3-mercaptohexanol (*d*_8_-3-MH), ^10^[H_2_]-4-mercapto-4-methyl-2-pentanone (*d*_10_-4-MMP), and ^5^[H_2_]-3-mercaptohexylacetate (*d*_5_-3-MHA), were synthesized based on protocols described elsewhere [35,36]. Model wine consisted of 12% *v/v* aqueous ethanol solution containing 7 g/L tartaric acid and the pH adjusted to 3.4 with 2 M NaOH. Water was 18 MΩ (Milli-Q, Millipore, Bedford, MA, USA).

### 3.2. GC-MS Conditions 

Quantification of the derivatized thiols during method optimization, method validation, and analysis of commercial samples was carried out by gas chromatography-electron impact-mass spectrometry (GC-EI-MS). Samples were analyzed with an Agilent 6890N GC coupled to an Agilent 5973N mass spectrometer (Santa Clara, CA, USA).

Optimization of extractive alkylation and HS-SPME conditions (Section 2.3) was performed on an Agilent DB-5 column (30 m × 0.25 mm × 0.5 μm) column. Samples were injected in splitless mode at 250 °C, and the split turned on after two minutes. The GC was operated at a constant pressure of 17 psi and helium was used as a carrier gas with an initial flow rate of 2 mL·min^−1^. The temperature program was: starting temperature of 50 °C with an initial ramp of 5 °C·min^−1^ up to 200 °C; then 20 °C·min^−1^ ramp to 250 °C, five minute hold.

Because of intermittent interferences and peak tailing on the DB-5 column for real wine samples, an Agilent DB-FFAP (30 m × 0.25 mm × 0.25 μm) column was used for determining figures of merit and analysis of real wine samples (Section 2.4 and Section 2.5). The GC was operated at a constant pressure of 10 psi and helium was used as a carrier gas with an initial flow rate of 1.2 mL·min^−1^. Samples were injected in splitless mode at 250 °C, and the split turned on after two minutes. The temperature program was: starting temperature of 50 °C with an initial ramp of 5 °C·min^−1^ up to 225 °C; then 20 °C·min^−1^ ramp to 250 °C, five minute hold.

The MS data was collected using selective ion monitoring (SIM) for specific time intervals. The quantifying ion, qualifying ions, and retention time for each compound and its corresponding deuterated standard are listed in Table 4. Data processing was carried out by Agilent Enhanced ChemStation v. E2.01 software.

**Table 4:** Retention times and quantification and qualification ions for thiol–pentafluorobenzyl (PFB) derivatives and their internal standards.

Compound	Retention Time (min)	Quantifying Ion	Qualifying Ions
4-MMP	26.0	*m*/*z:* 312	*m*/*z:* 181
*d*_10_-4-MMP	25.8	*m*/*z:* 322	*m*/*z:* 181
3-MH	31.7	*m*/*z:* 314	*m*/*z:* 181
*d*_8_-3-MH	31.5	*m*/*z:* 322	*m*/*z:* 181, 229
3-MHA	28.7	*m*/*z:* 227	*m*/*z:* *
*d*_5_-3-MHA	28.6	*m*/*z:* 255	*m*/*z:* 117

* lack of qualifier ions due to interferences for most *m*/*z* fragments of the compound.

### 3.3. Optimization of Sample Preparation Conditions

#### 3.3.1. Overview

A schematic overview of the thiol analysis method is shown in Figure A1, and steps subjected to optimization are underlined. All optimization experiments were performed on a model wine matrix. Descriptions of the optimized parameters, along with the values investigated, are listed in Table 5.

**Table 5:** Description and range of optimized derivatization and headspace solid phase microextraction (HS-SPME) parameters.

Parameters	Values	Description
*Derivatization Parameters*		
Agitation time ^a^	10, 25, 40 min	Agitation time during extractive alkylation
Solvent Volume ^a^	8, 12, 16 mL	Volume of organic solvent used for the extractive alkylation
pH ^b^	6, 9.5, 12	Sample pH following adjustment with 2 M NaOH
Catalyst ^b^	0, 0.05, 0.1 g	Amount of phase transfer catalyst (18-crown-6 ether)
Volume ^b^	10, 25, 40 mL	Volume of wine sample
*HS-SPME Parameters*		
Reconstitution Volume ^a^	0, 5, 10 mL	Volume of 17% *w*/*w* NaCl solution added to the dried down SPME vial prior to HS-SPME analysis
Time ^c^	10, 30, 60 min	HS-SPME extraction time
Temperature ^c^	50, 70, 90 °C	HS-SPME incubation and extraction temperature

^a^ Optimized value determined individually; ^b^ Optimized value determined as part of 3-factor CCF model; ^c^ Optimized value determined as part of 2-factor CCF model.

Because of the large number of parameters optimized, it was necessary to fix values for some parameters while optimizing conditions for other parameters. These are listed in Supplementary Table A1. Thiol concentrations in the model wines (3-MH, 3-MHA, 4-MMP) were adjusted among optimization experiments to achieve acceptable signal-to-noise ratios for each experiment; these concentrations are also listed in Supplementary Table A1. Optimal conditions were defined as those that resulted in the lowest detection limit. Limits of detection for each sample were calculated as 3 × noise, where the noise was estimated as the peak-to-trough height of a baseline interval adjacent to the peak by ChemStation (Agilent).

#### 3.3.2. Optimization of Sample Preparation Conditions

Five derivatization parameters were investigated: agitation time, organic solvent volume, sample pH, sample volume, and concentration of the 18-crown-6 ether phase transfer catalyst (Table 5). Three factors (sample pH, sample volume, phase transfer catalyst [PTC] concentration) that were suspected to have interaction terms were investigated using a center composite face-centered (CCF) model. Following determination of optimal conditions for these three factors, the remaining two factors—agitation time and solvent volume—were optimized individually because the former was not expected to interact with other parameters and the latter was too challenging to optimize in a CCF model because of its effects on evaporation time and vial size.

After optimization of derivatization conditions, three HS-SPME parameters were optimized—buffer reconstitution volume, extraction time and extraction temperature. Buffer reconstitution volume was optimized independently first, and then HS-SPME extraction time and temperature were examined by a CCF model.

#### 3.3.3. Optimization of Sample Extraction Method

In a 60 mL screw cap glass vial, 40 mL of wine was spiked with 80 µL of a *d*_10_-4-MMP standard solution ^10^[H_2_]-4-mercapto-4-methyl-2-pentanone (0.27 mg/L in 2-propanol), 80 µL of a *d*_8_-3-MH standard solution of ^8^[H_2_]-3-MH (0.22 mg/L in 2-propanol), and 80 µL of a *d*_5_-3-MHA standard solution (0.20 mg/L in 2-propanol). The pH of the mixture was adjusted using a 2 M solution of NaOH to a pH of 12 (±0.1) and 30 µL of the derivatizing agent (100 µL of 100% pentafluorobenzyl bromide (PFBBr) in 5 mL of 2-propanol) was added. The organic solvent (1:3 *v*/*v* pentane:diethyl ether, 9 mL) was added, and the sample was agitated on a shaker table for 10 min at room temperature (The Belly Dancer^®^ Hybridization Water Bath, Stovall Life Science, Greensboro, NC, USA). To break the resulting emulsion, the mixture was transferred to a centrifuge tube and centrifuged for 5 min at 13,000 rpm. The organic layer was transferred to a 20 mL SPME autosampler vial. Avoiding transfer of the aqueous layer or any emulsion was critical to avoid lengthy dry down times.

#### 3.3.4. Optimization of HS-SPME Analysis Conditions

The organic solvent was evaporated under N_2_ at room temperature. Near complete removal of solvent was critical, as residual hexane resulted in a loss of signal. Prior to HS-SPME, 10 mL of H_2_O and 2 g of NaCl were added to each vial. HS-SPME analyses were performed by a CombiPal system (Cohesive Technologies, Alpharetta, GA, USA). A 1 cm, 65 µm, SPME fiber (PDMS-DVB) was used for all experiments (Supleco, Bellafonte, PA, USA). The sample was extracted for 60 min at 70 °C and the compounds were then desorbed from the fiber directly in the GC injector in splitless mode for five minutes at 250 °C. The GC-MS operating conditions were described in Section 3.2.

### 3.4. Method Validation

#### 3.4.1. Linearity and Limits of Detection

Stock solutions of undeuterated and deuterated standards were prepared volumetrically in 2-propanol and stored at −4 °C until required. Calibration curves were generated by an eight-point standard addition for 4-MMP, 3-MH, and 3-MHA over a range of 6–20,000 ng/L in a model wine solution. Samples were prepared in duplicate according to the optimized method described above. Weighted linear regressions (1/x) of [312]/[322] ions *vs*. 4-MMP concentration, [314]/[322] ions *vs.* 3-MH concentration, and [227]/[255] ions *vs.* 3-MHA concentration were determined. Limits of detection were calculated from the same experimental data by the method of Pallesen [23].

#### 3.4.2. Accuracy

Accuracy was evaluated by recovery spikes using a commercially purchased 2011 California Pinot grigio selected for its relatively low endogenous thiol concentrations. The wine samples were spiked with either a low (60 ng/L) or high (500 ng/L) concentration of each thiol. Recovery experiments were performed in duplicate for each concentration.

#### 3.4.3. Precision

Precision was determined by performing replicate spikes into the Pinot grigio used in the accuracy experiments. The wine was spiked at high and low concentrations: 10 ng/L and 100 ng/L for 4-MMP, 25 ng/L and 200 ng/L for 3-MH, and 20 ng/L and 200 ng/L for 3-MHA. Five replicates were performed for each concentration, and precision was calculated as the relative standard deviation.

### 3.5. Quantification of Thiols in Commercial Wines

We quantified the three thiol concentrations in California (31) and New York (30) wines. Upstate New York wines were obtained in January and February 2013. Six types of wines (*n* = 5 for each) were included, of which five were labeled as varietal wines (Riesling, Sauvignon blanc, Gewürztraminer, Cayuga White, and Niagara).The sixth category was rosé wines produced from blends of red *V. vinifera* grapes (Cabernet franc, Lemberger, Cabernet Sauvignon, and/or Pinot noir). The wines ranging in vintages from 2007 to 2012. Following purchase, wines were stored at 10 °C until thiol measurements were performed in March 2013. For varietal California wines, commercial Cabernet Sauvignon (*n* = 20) and Sauvignon blanc (*n* = 11) wines were sourced in November 2014 and January 2015, respectively. Represented grape growing regions included Napa Valley (*n* = 13), Sonoma County (*n* = 9), Central Coast (*n* = 9), and Lodi (*n* = 1), and ranged in vintage from 2011 to 2013. Wines were stored at 4 °C until thiol measurement in February 2015.

### 3.6. Statistical Analyses

For the optimization experiments, CCF design-of-experiment and linear regression were performed on JMP version 9 (SAS Institute, Cary, NC, USA). For comparison of polyfunctional thiol concentrations among different wines, ANOVA and Tukey multiple-means tests were performed using JMP 9.

## 4. Conclusions

We have developed an optimized method for quantification of PFB derivatives of key polyfunctional thiols in wine (4-MMP, 3-MH, and 3-MHA). The method achieves good linearity and precision, and detection limits at or approaching the sensory thresholds for each thiol while utilizing commonplace GC-EI-MS instrumentation. Sample preparation was streamlined and detection limits improved by extractive alkylation followed by evaporation of the organic layer and back-addition of aqueous NaCl prior to HS-SPME extraction. We also report the first characterization of these thiols in wines from specific US wine regions (California and New York), as well as the first characterization in wines from interspecific hybrids, and generally observed lower concentrations of 3-MH/3-MHA and comparable or higher concentrations of 4-MMP as compared to the same varietal white wines from other regions.

## Appendix

**Table A1:** Conditions used for optimization of derivatization and HS-SPME parameters.

Parameter(s) Optimized	Model Wine Volume	Thiol Concentration	No. of Replicates	Constant Parameters
Agitation time	20 mL	200 ng/L	3	10 min extraction at 90 °C, pH 10, 0.1 g PTC, 4 mL solvent, 10 mL buffer
Solvent Volume	40 mL	3000 ng/L	3	60 min extraction at 70 °C, pH 12, 0 g PTC, 10 mL buffer, 10 min agitation
Reconstitution Volume	40 mL	200 ng/L	2	60 min extraction at 70 °C, pH 12, 0 g PTC, 9 mL solvent, 10 min agitation
pH, Volume, Catalyst	--	2000 ng/L	2	10 min extraction at 90 °C, 10 min agitation, 9 mL solvent
SPME Time, Temperature	40 mL	3000 ng/L	2	pH 10, 0.1 g PTC, 10 min agitation, 9 mL solvent
